# Genome-Wide Identification and Characterization of Gene Families in *Arachis*: Methods and Strategies

**DOI:** 10.3389/fgene.2020.00525

**Published:** 2020-05-27

**Authors:** Yongli Zhang, Dongmei Yin, Hui Song

**Affiliations:** ^1^Grassland Agri-Husbandry Research Center, College of Grassland Science, Qingdao Agricultural University, Qingdao, China; ^2^College of Agronomy, Henan Agricultural University, Zhengzhou, China

**Keywords:** *Arachis*, gene family, evolution, expression, homology

To date, at least eight *Arachis* genomes have been completely sequenced, including two *Arachis duranensis*, two *Arachis ipaensis*, one *Arachis monticola*, and three *Arachis hypogaea*. These datasets can provide a powerful starting point to understand the evolution of *Arachis* species. In addition to a comparison of *Arachis* species at the whole-genome level, evolutionary masks can be uncovered based on the analysis of *Arachis* gene families. Although many gene families have been identified and characterized in *Arachis*, different methods and strategies have been used by different researchers. This paper offers advice on the methods and strategies for identification, nomenclature, and quantitative real-time PCR (qRT-PCR) primer-design based on published datasets of *Arachis* gene families. The presented analyses provide a theoretical foundation for the improvement of the identification and characterization of gene families in *Arachis*.

## Genome Sequencing and Identification of Gene Families in *ARACHIS*

The cultivated peanut (*A. hypogaea*, AABB genome) was formed by the crossing of two wild peanuts: *A. duranensis* (AA genome) and *A. ipaensis* (BB genome) (Bertioli et al., 2016, 2019). In 2014, the genome sequences of *A. duranensis* (V14167) and *A. ipaensis* (K30076) were released on PeanutBase (https://peanutbase.org); however, their datasets were not usable at the time because the related paper had not been published then. It was not until 2016 when researchers could begin to use the datasets once the paper was finally published in Nature Genetics (Bertioli et al., 2016). In addition to these two lines, researchers sequenced two other lines: *A. duranensis* (PI 475845) and *A. ipaensis* (ICG_8206) (Chen et al., 2016; Lu et al., 2018). The genome sequences of three cultivated peanut species, namely *A. hypogaea* cv. Tifrunner, *A. hypogaea* cv. Shitouqi, and *A. hypogae*a cv. Fuhuasheng, were sequenced and released in 2018 (Bertioli et al., 2019; Chen et al., 2019; Zhuang et al., 2019). Simultaneously, the genome of a wild tetraploid peanut, *A. monticola*, was completely sequenced (Yin et al., 2018, 2019). These eight available genomic datasets provide raw material for the study of *Arachis* evolution.

Several researchers have focused on genome-wide analyses of the evolution and expression of gene families with canonical domains in *Arachis*. The WRKY transcription factor, a ~60-residue DNA-binding domain containing a conserved heptapeptide motif WRKYGQK, was first identified after the *A. duranensis* and *A. ipaensis* genomes had been released (Song et al., 2016b). Subsequently, aquaporin (AQP), basic/helix-loop-helix (bHLH), basic leucine zipper (bZIP), EXP (expansin), heat shock transcription factor (HSF), lipoxygenase (LOX), mildew resistance locus (MLO), nucleotide-binding sit–leucine-rich repeat (NBS–LRR), and phosphatidyl ethanolamine-binding protein (PEBP) gene families were identified in the *A. duranensis* (V14167) and *A. ipaensis* (K30076) genomes (Rispail and Rubiales, 2016; Song et al., 2016a, 2017; Gao et al., 2017; Guimaraes et al., 2017; Wang et al., 2017, 2019; Jin et al., 2019; Shivaraj et al., 2019) (Table S1). Growth-regulating factor (GRF) and NBS–LRR gene families were identified in the *A. hypogaea* cv. Tifrunner genome (Song et al., 2019; Zhao et al., 2019) (Table S1). However, different methods and strategies were used for the identification of gene families in *Arachis*.

## Identification Method of Gene Families in *ARACHIS*

At least three methods can be used to identify the members of a gene family. The first method identifies members based on gene annotations. The gene annotation that was generated based on reference genomes was added to the gene name. A gene family was identified using each gene name. This method requires more time when the larger genome is used. In addition, if the gene annotation is wrong, false-positive sequences emerge. The second method identifies members based on local BLAST (PSI-BLAST) or searches tool data from a public database (i.e., PeanutBase). Query sequences always originate from *Arabidopsis thaliana, Medicago truncatula*, and *Glycine max*. This method may lose particular gene family members because of species-specific genes. However, this method plays an important role for the identification of gene families with non-canonical domains. The third method identifies members based on a hidden Markov model (HMM) using the HMMER program (Finn et al., 2011). The HMM file was generated by a gene family from various organisms. HMM-based methods can provide an even better representation of gene families and allow the identification of more distant family members.

**Figure 1:**
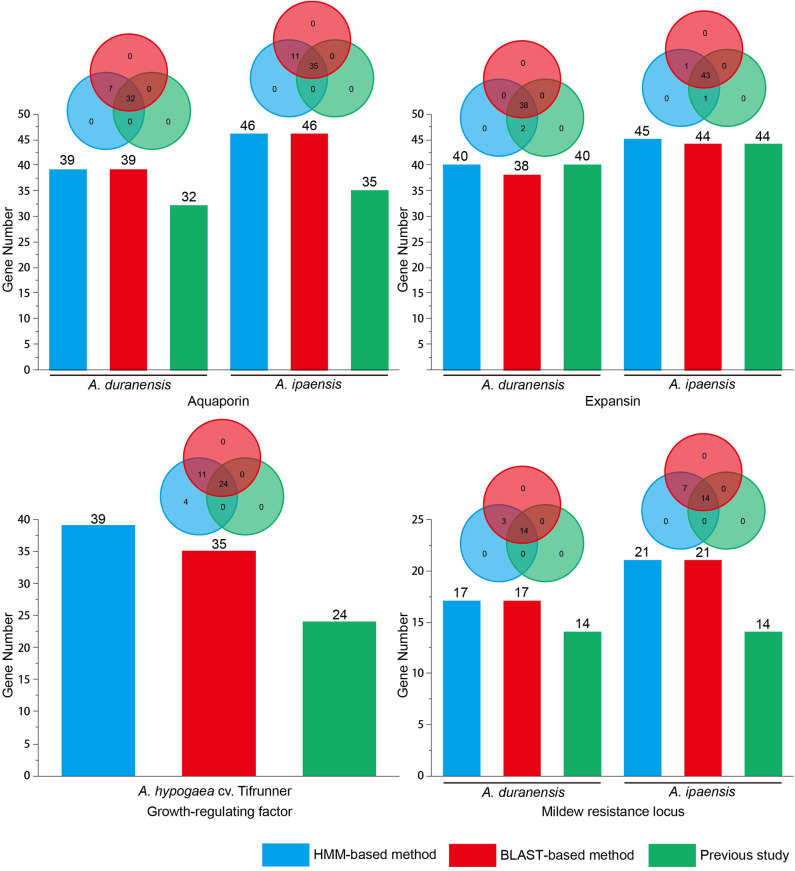
Identification of gene families in *Arachis* using HMM-based and BLAST-based method. Previous aquaporin data from Shivaraj et al. (2019); Previous expansin data from Guimaraes et al. (2017); Previous growth-regulating factor data from Zhao et al. (2019); Previous mildew resistance locus data from Rispail and Rubiales (2016).

A total of 12 gene families with canonical domains have been identified in *Arachis* (Rispail and Rubiales, 2016; Song et al., 2016a, 2017, 2019; Gao et al., 2017; Guimaraes et al., 2017; Wang et al., 2017, 2019; Jin et al., 2019; Shivaraj et al., 2019; Zhao et al., 2019). However, researchers used different methods to identify members among these gene families, specifically BLAST-based (four gene families) and HMM-based (eight gene families) methods (Table S1). Previous studies have demonstrated that more WRKY gene family members could be identified using the HMM-based method than the BLAST-based method in legumes (Song et al., 2018). To evaluate this result in various *Arachis* gene families, four gene families (AQP, EXP, MLO, and GRF) that were detected using the BLAST-based method in previous studies were re-identified using a HMM-based method. Previous studies identified gene families using different E-value thresholds (Table S2). If a smaller E-value was set, a smaller number of gene family members was obtained in the BLAST-based and HMM-based methods. For the PSI-BLAST and HMM programs, the default E-value parameter was 10. To compare the number of identified gene family members that used BLAST-based and HMM-based methods, this study used an E-value of 10 to re-identify the above-mentioned gene family members in *Arachis*. To verify the gene family domain, the obtained sequences were submitted to the Pfam database. The sequence was considered a gene family member if it contained a gene family domain. The obtained results showed that more members were identified using the HMM-based and BLAST-based method with an E-value of 10 than previous studies that used the BLAST-based method with an E-value below 10 among the above-mentioned four gene families (Figure 1 and Table S2). All members from the BLAST-based method were found in the HMM-based methods (Figure 1 and Table S3). In addition to this, compared with the BLAST-based method, the HMM-based method can identify a stable number of gene family members under an E-value of 10 in *Arachis*. Using *A. thaliana, Orazy sativa*, and *G. max* AQP and GRF gene family members to query against the *Arachis* genome for identification of a corresponding gene family in BLAST-based method, the same number of gene family members were detected using both the HMM-based and BLAST-based methods in AQP. However, a larger number of gene family members was detected using the HMM-based method than that when the BLAST-based method was used in GRF. In MLO, *A. thaliana* MLO was used as query sequence to identify gene family members in *A. duranensis* and *A. ipaensis*. The results showed that the same number of gene family members was detected using both HMM-based and BLAST-based methods. Nevertheless, more false positive sequences were found in BLAST-based method rather than HMM-based method (Table S4). To obtain more gene family members, multiple queries from different plants were considered when the BLAST-based method was used to identify gene families. However, if using the HMM-based method to identify gene families, the query sequence only selected the HMM file. Therefore, the HMM-based method is rapid and accurate. In summary, this study proposes that the best way to identify gene families in *Arachis* is the HMM-based method.

## Nomenclature for *ARACHIS* Gene Family Members

The nomenclature for *Arachis* gene family members could be classified into three types (Table S1). In the *Arachis* expansin gene family, *A. thaliana* expansin was used as reference (Guimaraes et al., 2017). In brief, the nomenclature for *A. thaliana* expansin was completed based on a chronological order of their discovery and phylogenetic tree (Kende et al., 2004). Synteny was constructed between *Arachis* and *A. thaliana* expansin. In four gene families (bHLH, LOX, and PEBP in *A. duranensis* and *A. ipaensis*; and NBS–LRR in *A. hypogaea* cv. Tifrunner), no nomenclature was allotted for members of gene families. The sequencing ID was used as gene name. In seven gene families (AQP, bZIP, HSF, NBS–LRR, MLO, and WRKY in *A. duranensis* and *A. ipaensis*; and GRF in *A. hypogaea* cv. Tifrunner), the nomenclature for members was defined by their chromosomal order. *Arachis duranensis, A. ipaensis*, and *A. hypogaea* cv. Tifrunner were referred to as Ad, Ai, and Ah, respectively. Following this procedure, the gene family name was listed and the number was then assigned based on the gene location in chromosomal order (e.g., AdWRKY1 and AdWRKY2). However, if a new member was found after the nomenclature had been assigned to a given gene family, the gene order of the new member should come after the last number of the legacy version.

## Identification of Duplicated Genes in *ARACHIS* Gene Families

Gene duplication is one of the driving forces of evolution and is a potential strategy for the adaptation to environmental change (Panchy et al., 2016; Van de Peer et al., 2017). To date, nine gene families were used to conduct homolog (paralog and ortholog) relationship analysis (Table S1). However, different methods were used to identify homology in *Arachis*, including phylogenetic tree, BLAST-based methods, and synteny relationship methods (Rispail and Rubiales, 2016; Song et al., 2016a,b, 2017, 2019; Guimaraes et al., 2017; Wang et al., 2017, 2019; Jin et al., 2019). Although these methods have been used to identify homologs in many studies, detailed parameters need to be listed. For example, which model was used and which bootstrap was credible for clades in the phylogenetic tree? Which threshold value was set for the synteny analyses? This paper recommends that researchers should consider using the BLAST-based homolog identification method in *Arachis* because this method has been verified for the identification of homologs in the cultivated peanut (Clevenger et al., 2016; Bertioli et al., 2019; Chen et al., 2019; Zhuang et al., 2019). The following evaluation criteria were used as thresholds to determine homology: (1) alignment coverage exceeding 80% of the two sequences; (2) identity > 80%; and (3) E-value ≤ 1E−10.

Gene completeness is a crucial factor that affects evolutionary analysis. Confusing results can be obtained when partial sequences are used in gene structure analysis because of the potential loss of introns and exons. In addition, selection pressure cannot be identified when partial homolog sequences are used. Therefore, it is suggested that full-length sequences of *Arachis* gene family members should be used for the evolutionary analyses. In addition to this, it is also worth noting that pseudogenes were identified during analysis of gene families. Although pseudogenes may play a crucial role in plant development and response to stress, most pseudogenes cannot code for proteins or loss of the original function. Therefore, pseudogenes were excluded when the selective pressures were estimated. In *A. duranensis* and *A. ipaensis*, CDSs with premature codons were reported in MLO, NBS–LRR, and WRKY gene families, which have been considered pseudogenes (Rispail and Rubiales, 2016; Song et al., 2016b, 2017).

## qRT-PCR Primer Design for *ARACHIS* Gene Families

The cultivated peanut is allotetraploid and contains many homologs. In addition, the members of gene families contain conserved sequences. Therefore, qRT-PCR primers are difficult to design because of non-specific amplification. Before the cultivated peanut genome was released, qRT-PCR primers were designed using the sum of *A*. *duranensis* and *A*. *ipaensis* sequences as cultivated peanut genome (Song et al., 2016a, 2017). Researchers focused on a problem to avoid the amplification of homologous sequences when designing the qRT-PCR primers in *Arachis* NBS–LRR and LOX gene families (Song et al., 2016a, 2017). Until now, the cultivated peanut genome can be used to study the expression of gene families. Future study has to carefully design the qRT-PCR primers to avoid non-specific amplification. The qRT-PCR primers are designed using the CDS with untranslated region (UTR) sequence because the UTR contained non-conserved sequences. Non-conserved regions are identified using multiple sequence alignment before designing the qRT-PCR primers. The Beacon Designer program was used for designing qRT-PCR primers. Beacon Designer can upload the genome sequence as a database. When a pair of qRT-PCR primers is designed, the program searches the database and lists the amplified fragment. This function can help researchers to remove false-positive primers.

## Conclusions

With the released *Arachis* genome sequence, more gene families can be identified and characterized. This study offers advice on gene family identification and characterization in *Arachis*. The HMM-based method can be used to identify members of a given gene family. Full-length sequences were used for evolutionary analysis. Homologs can be identified by a BLAST-based method. Non-specific amplification can be avoided in qRT-PCR.

## Author Contributions

HS and YZ conceived the study. HS wrote the paper. HS and DY approved the final version.

## Conflict of Interest

The authors declare that the research was conducted in the absence of any commercial or financial relationships that could be construed as a potential conflict of interest.

## Supplementary Material

Table S1 Identification of 12 gene families in *Arachis*.

Table S2 Comparison of four *Arachis* gene families using HMM-based and BLAST-based methods. ^a^The E-value was set to 10 in both the HMM-based and BLAST-based methods.

Table S3 Gene names in the four *Arachis* gene families. ^a^The E-value was set to 10 in both the HMM-based and BLAST-based methods.

Table S4 False positive rates in HMM-based and BLAST-based methods.
